# The genomic architecture of local adaptation in two connected populations of three-spined stickleback

**DOI:** 10.1093/g3journal/jkag086

**Published:** 2026-04-01

**Authors:** Sann Delaive, Nicolas Derôme, Sam Yeaman

**Affiliations:** Département de Biologie, Institut de Biologie Intégrative et Des Systèmes (IBIS), Université Laval, Québec, Quebec, Canada G1V 0A6; Département de Biologie, Institut de Biologie Intégrative et Des Systèmes (IBIS), Université Laval, Québec, Quebec, Canada G1V 0A6; Department of Biological Sciences, University of Calgary, Calgary, Alberta, Canada T2N 1N4

**Keywords:** recombination rate, structural variants, local adaptation, population genomics, stickleback

## Abstract

Populations often adapt to their local environments despite the homogenizing effects of gene flow, but the genomic mechanisms enabling this process remain unclear. Theory predicts that adaptive divergence under high connectivity is favored when beneficial alleles cluster in regions of reduced recombination, a pattern that can be reinforced by structural variants (SVs). We investigated this in three-spined sticklebacks (Gasterosteus aculeatus) from the St. Lawrence Estuary, where distinct freshwater and marine ecotypes meet and interbreed along a short ecological gradient. Using long- and short-read whole-genome sequencing, we mapped fine-scale recombination landscapes, cataloged SVs, and examined their relationship with adaptive genomic regions. Recombination landscapes differed between populations, with population-specific shifts in recombination rate estimated by an LD-based method. Putatively adaptive regions were not confined to low-recombination regions, yet SVs (inversions, insertions, and deletions) frequently coincided with local recombination suppression and elevated differentiation, suggesting they may contribute to local adaptation. Differentiated regions also overlapped disproportionately with previously-identified regions involved in repeated local adaptation across the species range, which tended to be strongly enriched on chromosomes IV, VII and XXI. These repeated regions were associated with lower recombination rates, suggesting that recombination suppression may contribute to their reuse across populations. As found in stickleback populations from other regions, the St. Lawrence populations exhibit elements suggestive of concentrated architectures clustered in a few genomic regions, along with relatively diffuse patterns of highly differentiated regions distributed genome-wide, across a wide range of recombination rates. These results highlight the intertwined roles of recombination variation and structural variation in shaping evolutionary trajectories in connected populations.

## Introduction

Understanding how populations adapt to their local environments remains a central question in evolutionary biology. Local adaptation occurs when resident genotypes exhibit higher fitness in their native environments compared to non-resident genotypes (Kawecki and Ebert 2004). This process generates intraspecific diversity, potentially enhancing a species’ evolutionary potential in the face of environmental change (Hu et al. 2021).

Although driven by natural selection, local adaptation can be constrained by gene flow, which homogenizes allele frequencies between populations and replaces adaptive alleles with neutral or even locally maladaptive alleles (Feder et al. 2012). Yet, empirical evidence shows that local adaptation can persist even under high gene flow (Limborg et al. 2012; Pespeni and Palumbi 2013; Muir et al. 2014). For instance, Trinidadian guppies have maintained locally adapted phenotypes despite repeated introductions of individuals experiencing different predation regimes (Fitzpatrick et al. 2015). Cases such as this raise a key question: which mechanisms might enable local adaptation to persist despite ongoing gene flow?

One explanation lies in the genomic architecture of adaptation, which encompasses the number, effect size, location, and linkage of adaptive loci. Under high gene flow, theory predicts that selection should favor clustered architectures in which adaptive alleles are tightly linked and thus inherited together (Yeaman and Whitlock 2011; Yeaman 2022). Recombination, which reshuffles alleles and breaks linkage, plays a central role in shaping these architectures. By creating new combinations of alleles, it can facilitate local adaptation in all sexual organisms (Smukowski and Noor 2011) or favor the creation of new mutations, which is favorable in a heterogeneous environment (Huang et al. 2015). In case of local adaptation with gene flow, recombination can also disrupt favorable combinations of locally adapted alleles (Samuk et al. 2017). Thus, local adaptation is expected to be facilitated when recombination is locally reduced, preserving clusters of adaptive loci (Tigano and Friesen 2016; Peñalba and Wolf 2020). Identifying how and where recombination landscapes vary between populations in different evolutionary scenarios and assessing the role of such variation on the genomic architecture of local adaptation is required to better understand what enables local adaptation to persist in the face of gene flow.

Recombination rates vary widely across genomes and among populations, influenced by chromosomal features (e.g. relatively low in centromeres and higher near telomeric regions), GC content (Fullerton et al. 2001), epigenetic modifications (Shilo et al. 2015), and structural variation. In particular, large structural variants (SVs)—including inversions, insertions, deletions, and duplications—can locally suppress recombination by disrupting homologous pairing and crossover formation (Völker et al. 2010; Morgan et al. 2017) and by yielding unbalanced karyotypes in recombinants (Kirkpatrick 2010). As a result, SVs have emerged as strong candidates for facilitating local adaptation under gene flow (Jay et al. 2018; Wellenreuther et al. 2019).

Inversions, detectable through patterns of linkage disequilibrium (LD) or local PCA (Ma and Amos 2012; Mérot 2020; Euclide et al. 2024), have received particular attention, with clear examples of inversion-mediated local adaptation in plants (Soudi et al. 2023), insects (Mérot et al. 2021), fishes (Meyer et al. 2024), and mammals (Harringmeyer and Hoekstra 2022). However, the adaptive role of other SV types—such as copy-number variants (CNVs), including insertions, deletions, and duplications—is also increasingly recognized. For example, CNVs associated with thermal adaptation have been reported in Atlantic capelin (Cayuela et al. 2020), American lobster (Dorant et al. 2020), pika (Sjodin et al. 2024), and common ragweed (Wilson et al. 2025). Despite their importance, non-inversion SVs remain underexplored due to historical challenges in detection. Advances in long-read sequencing now allow comprehensive characterization of all SV types within a single study (Mérot et al. 2020; Lecomte et al. 2024), avoiding biases inherent to indirect detection methods (Noor and Bennett 2009).

The three-spined stickleback (*Gasterosteus aculeatus*) is a well-established model for studying local adaptation. Having repeatedly colonized freshwater habitats from marine ancestors across the Northern Hemisphere (Fang et al. 2018), sticklebacks exhibit repeated phenotypic and genomic changes associated with freshwater adaptation. Resources such as a high-quality reference genome (Nath et al. 2021), a pedigree-based recombination map (Rastas et al. 2016), and extensive ecological and genomic data (Hohenlohe and Magalhaes 2020) have enabled a detailed study of adaptive divergence between marine and freshwater populations. Freshwater adaptation in sticklebacks often displays a concentrated genomic architecture, characterized by a few large-effect loci embedded within “islands of differentiation,” such as the well-known *Eda* locus controlling lateral plate number (Jones et al. 2012; Roberts Kingman et al. 2021), as well as many smaller-effect loci scattered across the genome. These islands are often reused across independent freshwater colonization events (Hohenlohe et al. 2010; Jones et al. 2012; Roberts Kingman et al. 2021), likely because they contain alleles maintained as standing genetic variation in the ancestral marine population (Nelson and Cresko 2018). Multiple studies have also demonstrated the role of different structural variants in freshwater adaptation (Chain et al. 2014; Rodríguez-Fuentes et al. 2025). Moreover, Shanfelter et al. (2019) have demonstrated the potential for the recombination landscape to vary greatly between a freshwater and a saltwater population. All these characteristics make the three-spined stickleback a suitable species to study the relationship between recombination, structural variation, and their impact on the genomic architecture of local adaptation.

In this study, we analyzed long- and short-read whole-genome sequencing data from three-spined sticklebacks inhabiting the St. Lawrence Estuary. A previous study by Delaive et al. (2025) demonstrated the presence of two genetically distinct main populations in this system—a marine and a fluvial one—showing asymmetric gene flow (*m* = 0.0005) that has persisted for at least one thousand generations. This timeframe is sufficient for the evolution of local adaptation, particularly in sticklebacks, which are known to adapt rapidly to freshwater environments (Bell and Aguirre 2013; Lescak et al. 2015). The coexistence of two connected populations provides a good opportunity to investigate the genomic architecture of local adaptation in the presence of ongoing gene flow. We first tested whether observed recombination landscapes estimated based on variation in LD differ between freshwater and marine populations and identified where along the genome such differences are most pronounced. We then asked whether recombination landscape divergence and regions of suppressed recombination tend to coincide with elevated genetic differentiation. Finally, we evaluated whether SVs contribute to recombination differentiation between populations by analyzing their association with population-specific shifts in recombination rate. Together, these analyses provide new insights into how recombination and SVs interact to shape the genomic architecture of local adaptation in the face of gene flow.

## Materials and methods

### Study system

**Fig. 1.**
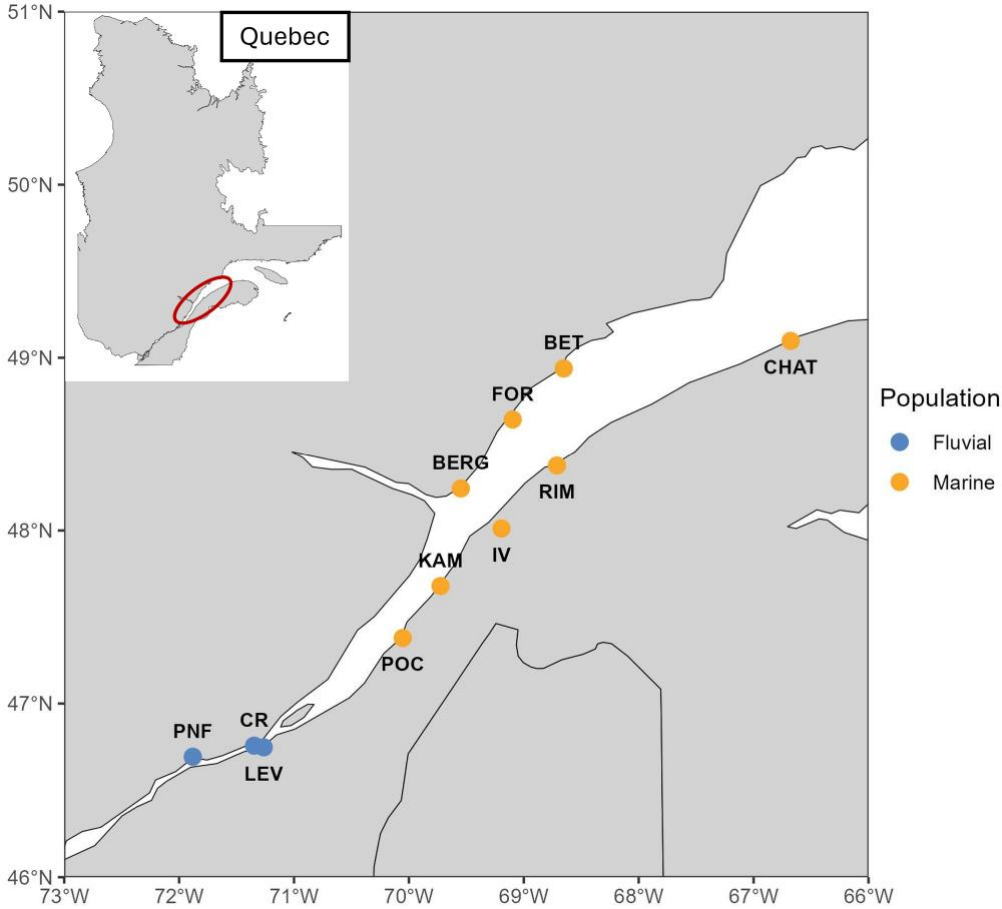
Sampling locations of three-spined sticklebacks (*Gasterosteus aculeatus*) in the St. Lawrence Estuary. A total of 40 individuals with a balanced sex ratio were collected from 8 sites in the marine and middle estuary, representing the marine population as defined in Delaive et al. (2025), and from 3 sites in the fluvial estuary, corresponding to the fluvial population. Sampling locations are distinguished by color according to population. Location abbreviations: PNF, Portneuf; CR, Cap-Rouge; LEV, Lévis; POC, La Pocatière; KAM, Kamouraska; IV, Isle-Verte; BERG, Grande-Bergeronnes; RIM, Rimouski; FOR, Forestville; BET, Betsiamites; CHAT, Cap-Chat.

We analyzed a subset of the whole-genome resequencing dataset previously published in Delaive et al. (2025). In brief, genomic DNA was extracted from fin tissue and sequenced on an Illumina NovaSeq 6000 platform (PE150), targeting ∼15× coverage per individual. After quality control, reads were aligned to the most recent stickleback reference genome (Nath et al. 2021). SNPs were called using bcftools mpileup (v.1.12). Sex chromosomes (chrXIX and chrY), mitochondrial DNA (chrM), and unplaced contigs (chrUn) were excluded from all analyses. SNPs with more than two alleles and a minor allele frequency <0.05 were filtered out. Additional filtering excluded SNPs with total sequencing coverage < 4 or >35, as well as SNPs genotyped in 90% of the individuals. The resulting dataset comprised 2,332,752 high-quality SNPs genotyped across 387 three-spined sticklebacks sampled along a salinity gradient in the St. Lawrence Estuary, Canada. To confirm the absence of population structure inside, we conducted an individual ancestry analysis using ADMIXTURE v.1.3.0, testing K values ranging from 2 to 10 (Alexander et al. 2009). The optimal K was K = 2 (CV error = 0.50227, Supplementary Fig. 1). We excluded individuals from Baie-Saint-Paul, which appeared genetically distinct and could bias comparisons between the primary marine and fluvial groups, as observed at K = 3 and as suggested by Delaive et al. (2025). We also removed individuals that clustered differently in an identity-by-missingness analysis conducted with PLINK (Chang et al. 2015). The final dataset thus consisted of 359 individuals: 89 from a fluvial population and 270 from a marine population. The fluvial population included individuals sampled from three sites in the fluvial estuary (low salinity), whereas the marine population encompassed individuals from eight sites distributed across the middle and marine sections of the estuary, where salinity progressively increases to ∼30 ppt (Fig. 1).

The generation of the dataset is described in greater detail in Delaive et al. (2025).

### Construction of recombination maps

We reconstructed fine-scale recombination maps using Pyrho (Spence and Song 2019), which models LD using fused-LASSO regression while incorporating historical changes in effective population size (Ne).To provide this information to Pyrho, population-specific Ne trajectories were estimated with SMC++ v1.15.4 (Terhorst et al. 2017), based on all individuals (Supplementary Fig. 2). VCF files were converted using *vcf2smc*, and replicate histories were combined into composite likelihoods to improve inference. Analyses assumed a mutation rate of 4.56 × 10^−9^ mutations per site per generation (Zhang et al. 2023) and a generation time of one year (Reid et al. 2021). Because demographic inference relies on patterns of heterozygosity and coalescent events, runs of homozygosity longer than 150 kb were masked before inference to avoid overestimation of Ne changes.

To account for within-population heterogeneity, especially in the marine group that encompasses a larger geographical area, we divided each population into two replicate subsets by randomly selecting 42 individuals. We repeated this process three times to create six subsets by population, all comprising a different combination of individuals. The fluvial population was fully included in all replicates, while the marine population subset replicates were drawn from a pool of 84 individuals sampled across six sites to compare similar sample sizes. This resulted in twelve recombination map subsets in total.

Recombination maps were generated using Pyrho following consistent hyperparameter settings across replicates and populations. Lookup tables, representing expected patterns of LD under a specified demographic model and a range of recombination rates, were generated using *–make_table* with a diploid sample size of 42 and a Moran size of 110. The Moran size defines the number of lineages used in the Moran model, a forward-time approximation of the coalescent with recombination, and affects the precision of LD expectations under a given demographic history (Kamm et al. 2016). Window size and block penalty parameters were optimized using –*hyperparam* by minimizing the L2 norm, defined here as the sum of squared differences between observed and expected LD values. The block penalty determines the smoothness of the recombination landscape by penalizing excessive changes in estimated rates between adjacent genomic windows. Based on this optimization, a block penalty of 15 was applied for all chromosomes, with an optimal window size of 90 SNPs for most chromosomes and 30 SNPs for chromosomes XI and XX. Recombination rates were then estimated with Pyrho's optimize function using the *–fast-missing* flag. Estimates were obtained separately for each of the six subsets per population, and chromosome-level outputs were concatenated to produce genome-wide, per-base, per-generation recombination maps for each subset.

Strong selection can locally increase LD around F_ST_ outliers, which may create circularity in LD-based recombination analyses. Because methods such as Pyrho interpret high LD as evidence of low recombination, this can downwardly bias recombination estimates and affect recombination-aware outlier detection. We therefore complemented our analyses with an independent, pedigree-based recombination map. Fine-scale recombination rates were extracted from the linkage map of Rastas et al. (2016), for which the coordinates were lifted on the latest three-spined stickleback reference genome (Sylvestre et al. 2023). To assess concordance between linkage map recombination rate estimates and Pyrho-inferred recombination rates, we computed chromosome-wise Pearson correlation coefficients between recombination rate in cM/Mb (from the linkage map) and mean population-scaled recombination rate (ρ) using the *cor.test* function in R.

### Comparisons of recombination maps

To quantify divergence in recombination landscapes between freshwater and marine populations, we applied two complementary metrics introduced by Talbi et al. (2025): the Population Recombination Divergence Index (PRDI) and a window-based measure of recombination rate dissimilarity, hereafter referred to as Δ*r*. While the PRDI quantifies the broad-scale divergence in recombination landscapes of two populations that cannot be explained by demographic differences alone, the Δr identifies fine-scale patterns of divergence based directly on recombination rate variation.

The PRDI is defined as the difference between two metrics: *me* (empirical divergence) and *ms* (simulated divergence under neutrality). To calculate it, raw recombination rates obtained from pyrho were first averaged in nonoverlapping 2 kb windows using the *bedtools map* function. We then assessed the consistency of recombination rate estimates within and between populations by computing pairwise Spearman correlations across subsets using 5 Mb sliding windows along each chromosome. For each window, we calculated all within-population correlations (among freshwater subsets and among marine subsets) and all between-population correlations (across freshwater and marine subsets). The metric *me* was defined as the difference between the lowest median in all within-population correlation and the median between-population correlation.

To establish a neutral expectation (*ms*), we performed coalescent simulations using msprime v1.3.3 (Baumdicker et al. 2022). We simulated 84 diploid individuals, partitioned into two groups of 42 to mimic freshwater and marine populations. Simulations assumed a shared recombination landscape across populations, parameterized using the empirically inferred freshwater recombination map (results were unchanged when using the marine map instead). Demographic parameters, including effective population sizes and split times, were informed by SMC++ estimates. For each simulated dataset, recombination maps were reconstructed using the same Pyrho pipeline applied to the empirical data. The *ms* metric was then calculated from the simulated maps following the same procedure as for *me*, and PRDI was defined as the difference between *me* and *ms*.

To further characterize regions where recombination divergence is more pronounced between freshwater and marine populations, we looked for 100 kb windows where the Manhattan distance between two subsets from different populations (between-population Δr) exceeds the Manhattan distance between two subsets from the same population (within-population Δr). Each Δr (within and between) was calculated as the pairwise Manhattan distances in non-overlapping 100 kb windows, each composed of 50 adjacent 2 kb bins. This Manhattan distance was then scaled by the number of bins, and log transformed to stabilize variance. For each 100 kb window, we calculated the median within-population and between-population Δr. Windows where the between-population Δr exceeded three standard deviations above the genome-wide mean of within-population Δr were considered Δr outliers.

Recombination rate varies along the genome and can happen relatively frequently in some regions (hotspots) and rarely in others (coldspots). To understand if these particular regions are conserved between populations, we identified recombination hotspots and coldspots by population using a threshold-based approach. For each window in each empirical recombination map, we computed the local background mean and standard deviation of log-transformed recombination rates within a ± 20 kb window. Windows exceeding three standard deviations above or below the local mean were classified as hotspots or coldspots, respectively. Adjacent significant windows were merged with a 1 kb maximum gap using bedtools *merge* to reduce redundancy. Hotspots longer than 5 kb were removed as they are subject to known bias (Hoge et al. 2024). We then used bedtools *intersect* to identify hotspots and coldspots consistently shared within and between populations. Two hotspots/coldspots were considered shared if their regions overlap by at least one base pair.

### Identification of highly differentiated genomic regions

We identified regions of elevated genetic differentiation between marine and fluvial populations by calculating per-SNP Weir and Cockerham's F_ST_ using the *–weir-fst-pop* function in VCFtools. F_ST_ values were aggregated into non-overlapping 100 kb windows, a size selected to balance SNP density and local resolution, given the rapid decay of LD estimated approximately to 1 kb in the three-spined stickleback genome (Roesti et al. 2015). Windows with less than ten SNPs were filtered out as they are particularly subject to false positives.

To control the confounding influence of recombination rate variation, we assigned each window to one of five recombination bins defined by the 0 to 20th, 20 to 40th, 40 to 60th, 60 to 80th, and 80 to 100th percentiles of the genome-wide recombination distribution. As discussed previously, the recombination bins were defined with the linkage map recombination rate instead of the Pyrho-inferred recombination rate to avoid circularity. Within each bin, we defined outlier SNPs as those exceeding the 99th percentile of F_ST_ values, allowing for recombination-informed detection of elevated differentiation without applying a uniform genome-wide threshold.

To further reduce SNP-level noise, we identified outlier windows as those with an enrichment of SNPs per window using a binomial framework. For each 100 kb window, we calculated the binomial probability of observing a given number of outlier SNPs or more, considering the total number of SNPs in the window and the genome-wide proportion of outliers. While non-independence among SNPs precludes formal *P*-value calculation, this enrichment test highlights windows containing disproportionate numbers of highly differentiated variants and controls for SNP density. Windows with a non-exact *P*-value < 0.05 were retained for downstream analyses. Adjacent outlier windows were subsequently merged into single continuous regions, and the deviation of the distribution of outlier windows across the five recombination bins from the null expectation of equal representation (20% per bin) was tested using an X^2^. test. To further ensure that outlier windows were not simply those with few SNPs, we also recorded the number of SNPs in each window and evaluated the correlation between SNP count and the non-exact *P*-value.

To test whether regions previously identified as repeated targets of local adaptation in global stickleback populations (Roberts Kingman et al. 2021) overlapped with outlier windows in our dataset, we first lifted over the coordinates of repeated regions to our reference genome using the snplift tool (https://github.com/enormandeau/snplift). Coordinates were manually curated to retain high-confidence mappings. We evaluated the enrichment of globally repeated regions within outlier windows in our study, using a permutation-based approach. Overlap was defined as any intersection between a 100 kb window and a repeated region. To determine whether the observed overlap was greater than expected by chance, we performed a chi-squared test comparing the number of overlapping and non-overlapping windows for both outlier and non-outlier sets. To account for genome structure and recombination heterogeneity, we also performed a permutation test. At each iteration (*n* = 500), the set of outlier windows was randomly repositioned across the genome while maintaining chromosome identity and window size. For each permutation, we recalculated the chi-squared statistic and generated a null distribution. An empirical *P*-value was then computed as the proportion of permuted chi-squared statistics greater than or equal to the observed value.

### Relationship between recombination and genetic differentiation

To investigate the link between recombination divergence and genetic differentiation, we evaluated the genome-wide association between windowed F_ST_ and PRDI using a Spearman correlation, calculated in non-overlapping 5 Mb windows. Correlation significance was assessed with the *cor.test* function in R.

Because variation in F_ST_ can also reflect underlying heterogeneity in nucleotide diversity, we modeled windowed F_ST_ as a function of recombination rate and diversity. Nucleotide diversity (π) was estimated separately in marine and fluvial populations using Pixy v.1.2.10.beta2 in 100 kb non-overlapping windows. A multiple linear regression by population was then used to test the effect of the linkage map recombination rates and π on F_ST_. We also tested the difference in the average level of π between populations using a Student's T-test.

To further explore the interaction between recombination and natural selection, we fitted a linear model of log-transformed ρ as a function of cM/Mb from the meiotic recombination map and extracted the residuals. These residuals represent windows where observed LD is higher or lower than expected given meiotic recombination, potentially reflecting the influence of evolutionary processes such as selection. We then tested whether these residuals were correlated with π in freshwater and marine populations, and with F_ST_ between populations, to evaluate whether regions of low diversity or high differentiation exhibit LD deviations. We also tested if globally repeated and non-globally repeated outlier regions had different residual values than the genomic background using multiple T-test.

### Structural variant catalogue and genotyping

We analyzed structural variation using an updated version of the SV catalogue established in Delaive et al. (2025), which combines long-read and short-read sequencing data. In the original study, long reads from Oxford Nanopore sequencing were aligned to the stickleback reference genome using Winnowmap v2.03. SVs were identified using three long-read callers (Sniffles2, SVIM, NanoVar) and three short-read callers (Delly, Manta, Smoove). Variants supported by at least two callers within each data type were retained, and long- and short-read sets were merged into a unified SV catalogue. This combined list of deletions, insertions, and inversions was genotyped across all individuals using VG Giraffe, a graph-based genotyping tool designed to account for complex structural polymorphisms. We filtered out all SVs with a MAF < 0.05 and that were genotyped in at least 50% of the individuals. For the present study, we retained the full set of insertions and deletions and excluded all inversions defined in the original catalogue.

Inversions were re-evaluated to improve call reliability. Initial inversion calls generated with Sniffles were manually curated following a protocol developed by Fantine Benoit (in prep.). Two of the 16 individuals sequenced with long-read data (BERG_23 and CHAT_02) were randomly selected for detailed inspection in IGV. This targeted validation strategy was chosen to assess inversion call accuracy while maintaining a feasible analysis workload.

For each selected individual, all detected inversions were visually inspected to evaluate read orientation, local coverage patterns, breakpoint clarity, and overlap with other SVs. Inversions lacking clear long-read support, exhibiting ambiguous breakpoints, or overlapping other rearrangements were classified as false positives and excluded from downstream analyses. Among the 230 inversion candidates examined, 43% were confirmed as true inversions based on manual curation. Consistent with these observations, nested and overlapping variants were further collapsed by retaining only the longest non-overlapping representative variant. This smaller curated inversion set, along with the previously validated insertions and deletions, was re-genotyped across all individuals using an updated genome graph genotyping pipeline (https://github.com/LaurieLecomte/genotype_SVs_SRLR_update.git).

### Analysis of structural variants

To identify SVs with elevated differentiation between habitats, we calculated F_ST_ by variant between marine and fluvial populations using VCFtools’ *–weir-fst-pop* function. Outliers were defined as SVs exceeding the 99th percentile of the F_ST_ distribution.

To test whether outlier SVs were preferentially located within highly differentiated genomic regions, we performed a Fisher's exact test based on their overlap with windows enriched for F_ST_ outlier SNPs. We then constructed a 2 × 2 contingency table contrasting the number of outlier and background SVs that overlapped at least one F_ST_-enriched window vs those that did not. The Fisher's exact test was implemented in R using the *fisher.test* function with a two-sided alternative hypothesis. Because this pattern may be influenced by SV length (Conrad and Hurles 2007), we stratified SVs into two size classes: large SVs (≥ 1 kb) and small SVs (< 1 kb). The same analysis was performed separately for each size class.

As a control analysis, we evaluated whether the relative density of SVs within windows impact the outcomes of genomic differentiation. SV density within each 100 kb window was quantified as the proportion of base pairs overlapped by SVs, treating deletions as 1 bp long. The relationship between SV density and enrichment significance was then assessed using Spearman's rank correlation between SV density and –log_10_(*P*-values).

We evaluated the impact of SVs on local recombination by modeling patterns of LD around SVs. Pairwise LD (r^2^) was estimated using the *–geno-r2* function in VCFtools, within 35,000 bp windows, separately for each chromosome and population. For each SV, we extracted a single SNP pair flanking the SV within ±5 kb and calculated three explanatory variables: the inverse of the frequency-weighted physical distance between SNPs, the frequency of the SV in the population, and the proportion of the interval between flanking SNPs covered by the SV.


$$Proportion\;covered\;=\;\frac{\left|Length_{SV}\right|}{Position_{SNP2}-Position_{SNP1}}$$

We fit linear mixed-effects models separately for the freshwater and saltwater datasets adding the SV type as another explanatory variable and the name of the chromosome as random effect. Both frequency-weighted distance and the flanking SNP interval were scaled prior to modeling. For each population, we modeled r^2^ as a function of scaled distance, proportion covered, and SV type, with chromosome included as a random effect to account for non-independence of observations within chromosomes. This approach allowed us to leverage the full dataset while controlling for chromosome-level structure. Models were fit in R using the lmer function from the lme4 package and estimated marginal means for SV types were compared using the emmeans package.We also tested if SVs were preferentially located in both shared or population-specific coldspots and/or hotspots of recombination. To assess enrichment, we counted the number of SVs overlapping cold and hotspots per category. To generate a null distribution, the positions of these genomic regions were randomly shuffled along each chromosome, preserving the original size distribution. We repeated this randomization 10,000 times per category. *P*-values were calculated as the proportion of permutations in which the number of overlapping SVs was greater than or equal to the observed count.

## Results

### Population-specific recombination maps

We reconstructed fine-scale recombination maps for marine and fluvial populations of three spined sticklebacks from the St. Lawrence Estuary using a LD-based approach. To contextualize recombination patterns, we first inferred historical changes in Ne using SMC++. Both populations showed signatures of slightly different demographic histories. In the fluvial population, a steep bottleneck is followed by two phases of population expansions, with contemporary Ne estimates per chromosome ranging from 30,005 to 114,882, with a mean of 88,986. The marine population showed a similar bottleneck but without a clear population expansion and contemporary Ne distribution, ranging from 12,431 to 20,450, with a mean of 16,140.

**Fig. 2.**
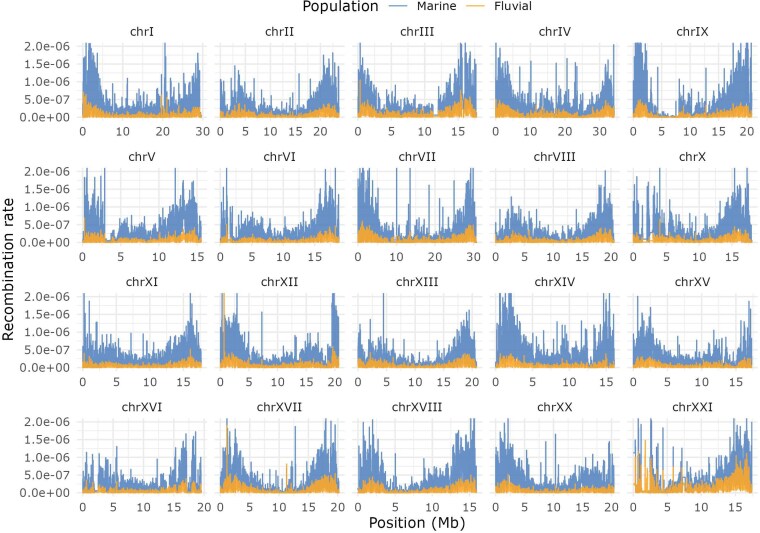
Genome-wide recombination rate variation in freshwater and marine three-spined stickleback. Recombination rates varied substantially across chromosomes and along their lengths in both populations. Each panel shows one chromosome, with the *x*-axis representing position in megabases (Mb) and the *y*-axis showing the per-base, per-generation recombination rate. Lines show the median recombination rate per 40 kb window, calculated across empirical replicates within each population. Recombination rates were capped at 2 × 10^−6^ to improve visualization. Colors denote marine and fluvial populations.

Genome-wide recombination rates varied substantially across chromosomes and along their lengths in both populations (Fig. 2). In the fluvial population, rates ranged from the 5th percentile of 1.65 × 10^−9^ per base per generation on chromosome XVII to the 95th percentile of 4.98 × 10^−7^ on chromosome XXI. In the marine population, values spanned from 4.51 × 10^−10^ on chromosome XVII to 1.362 × 10^−6^ on chromosome IX. To evaluate the similarity of these LD-based estimates to more direct estimates of meiotic recombination rate, we compared them with an independent pedigree-based linkage map (Rastas et al. 2016). Genome-wide recombination rates from both approaches were strongly correlated (Pearson's r = 0.62), with most chromosomes showing even higher concordance (r = 0.69 to 0.73). Notably, correlations were weaker (r ≈ 0.55) on chromosomes V, IX, XVI, and XXI.

### Divergence in recombination landscapes between populations

**Fig. 3.**
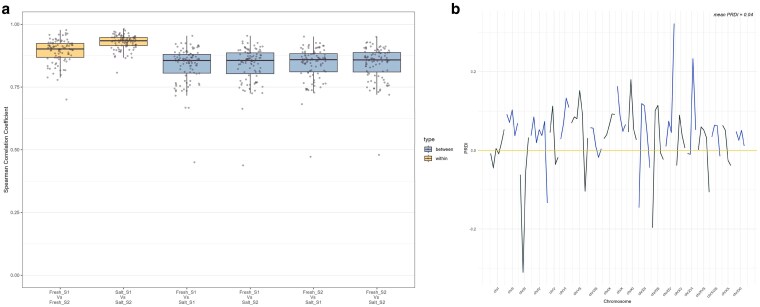
Differences in recombination landscape similarity between freshwater and marine three-spined stickleback. a) Boxplot representing pairwise Spearman correlations of recombination rates within and between replicate maps for fluvial (ρ = 0.91) and marine (ρ = 0.90) populations, and between populations (ρ = 0.80). Correlations were higher within populations than between them, indicating greater similarity among replicates from the same population. S1 and S2 indicate the two subsets analyzed per population. b) Variation in PRDI along the genome. Positive values indicate genomic regions with greater divergence in recombination landscapes between populations, whereas negative values denote conserved regions.

We assessed whether recombination landscapes differed between freshwater and marine populations by comparing recombination rate similarity within and between replicate maps. Pairwise Spearman correlations revealed higher consistency within populations (ρ = 0.90 in freshwater; ρ = 0.93 in marine) than between them (ρ = 0.86), indicating that recombination landscapes were more similar among replicates from the same population than across populations (Fig. 3a).

To determine whether this reduction in between-population correlation could be explained by different demographic history alone, we performed neutral coalescent simulations under a model assuming identical recombination landscapes in both populations. In simulated data, within-population correlations were slightly higher (ρ = 0.95 in freshwater; ρ = 0.93 in marine), and between-population correlation also increased (ρ = 0.88). The difference between empirical and simulated values was summarized using the Population Recombination Divergence Index (PRDI), yielding a genome-wide value of 0.04.

PRDI values varied along the genome, indicating spatial variation in recombination landscape divergence. Positive PRDI values indicate a difference in the recombination landscapes between populations while negative values point to more conserved regions. The highest value was observed on chromosome XIV (PRDI = 0.32), while the lowest occurred on chromosome III (PRDI = −0.31) (Fig. 3b).

To further quantify localized divergence, we computed pairwise recombination rate dissimilarity (Δ*r*) between freshwater and marine populations in non-overlapping 100 kb windows. This analysis identified only three windows with elevated between-population divergence relative to within-population variation: two adjacent windows on chromosome XVII and one on chromosome IV.

We next examined fine-scale recombination features by identifying hotspots and coldspots of recombination in each population. Using a deviation-based thresholding approach with a 40 kb background, we detected 105 recombination hotspots in the marine population and 188 in the fluvial population. Among these, 58 hotspots were shared between the two populations (19% overlap, representing 55% of marine and 31% of freshwater hotspots). Coldspots were similarly shared: 429 were identified in the marine population and 208 in freshwater, with 98 shared (15% overlap, representing 23% of marine and 47% of freshwater coldspots).

### Repeatable genomic differentiation between populations

To evaluate how recombination rate variation contributes to genetic differentiation, we identified 553 outlier windows enriched for highly differentiated SNPs using a recombination-informed framework. According to a X^2^ test, outlier windows were distributed evenly across five recombination bins (*P* = 0.66, Supplementary Fig. 3) and spanned 0.02% of the genome. We detected no significant correlation between the non-exact *P*-value and SNP density across 100 kb windows (Pearson's r = −0.009, *P* = 0.569, Supplementary Fig. 4).

**Fig. 4.**
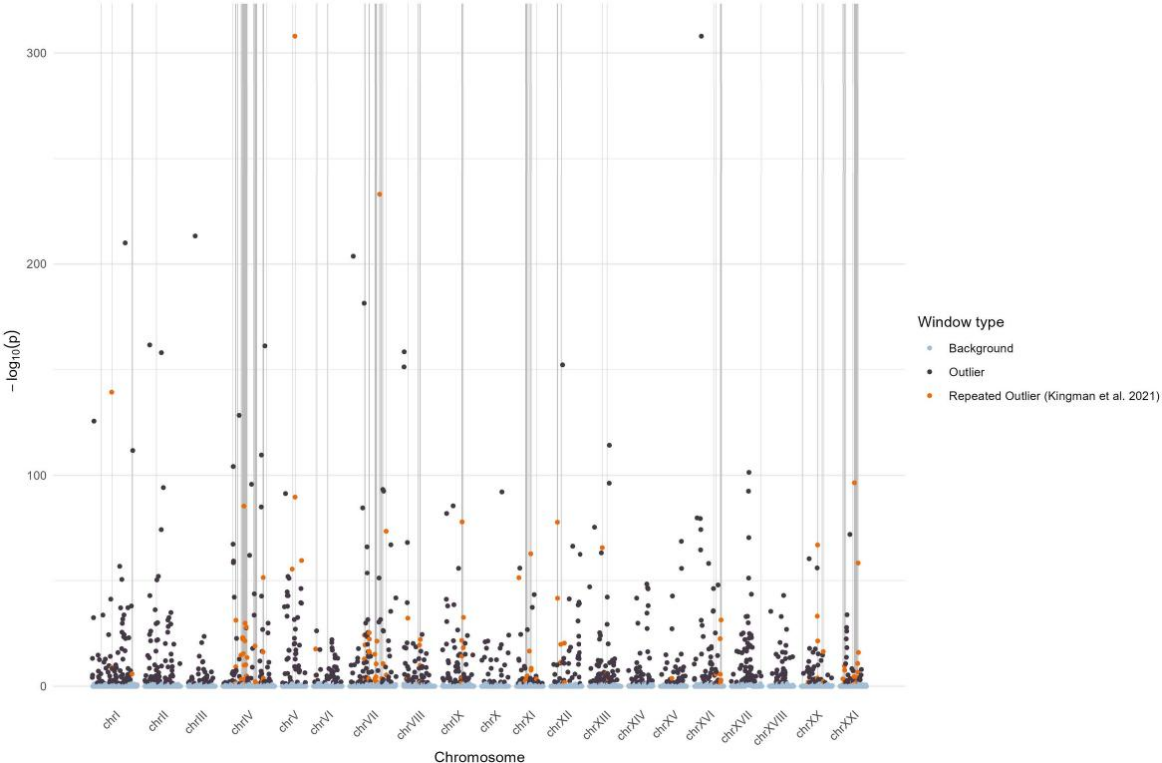
Overlap between enriched regions in our dataset and globally repeated targets of adaptation in three-spined stickleback. Each point represents a 100 kb genomic window, with light gray regions corresponding to the 92 adaptive regions identified by Roberts Kingman et al. (2021). Enriched windows are shown in black, and those located within highlighted regions, termed “repeated outliers”, are shown in orange. The y-axis represents the –log_10_-transformed non-exact *P*-value from a binomial test assessing enrichment of outliers in each window. Among 1,037 enriched windows, 71 (6.85%) overlapped at least one repeated region, and 54 of the 92 repeated regions (58.6%) contained at least one enriched window.

Moreover, we evaluated whether outlier windows in our population also coincide with previously identified genomic regions exhibiting repeated signatures of adaptation across the species range. To do that, we compared our 100 kb outlier windows with the 92 outlier regions identified by Roberts Kingman et al. (2021; hereafter “repeated region”). Among the 1,037 outlier windows identified in this study, 71 (6.85%) overlapped at least one repeated region (Fig. 4). Conversely, 54 of the 92 previously identified repeated regions (58.6%) contained at least one outlier window. This represents a more than twofold enrichment compared to background genomic windows, 3.03% of which overlapped with repeat regions (χ^2^ = 30.67, *P* < 0.001). A permutation test confirmed that this enrichment is unlikely to occur by chance, with none of the 500 permuted χ^2^ values exceeding the observed value (mean = 1.53, sd = 2.05, empirical *P* < 0.002).

### The interplay of recombination, nucleotide diversity and natural selection

To disentangle the interplay between recombination, π and natural selection, we evaluated the relationship between F_ST_ and recombination rate using complementary models. First, when looking at the relationship between F_ST_ values and PRDI, we observed a non-significant correlation between these two metrics at the scale of 5 Mb windows (Spearman ρ = 0.23, *P* = 0.102).

Second, to test if elevated F_ST_ may result from both directional selection and confounding factors such as linked selection or ancestral diversity differences, we modeled log-transformed F_ST_ as a function of recombination rate and within-population π separately for the freshwater and saltwater populations. Both models explained a similar proportion of F_ST_ variance (adjusted R^2^ = 0.134 for freshwater; 0.131 for saltwater, *P* < 2.2 × 10^−16^ in both cases), and in each case revealed a significant interaction between π and recombination rate (interaction estimates = −7.98 and −7.57, *P* < 1 × 10^−5^). These interactions indicate that the relationship between nucleotide diversity and genetic differentiation depends on the recombination landscape: specifically, the correlation between π and F_ST_ is stronger in regions of low recombination. At the genome-wide scale, π was slightly higher on average in the marine population (π = 0.00261) than in the fluvial population (π = 0.00255), but this difference was not significant (t = −1.8064, *P* = 0.07).

**Fig. 5.**
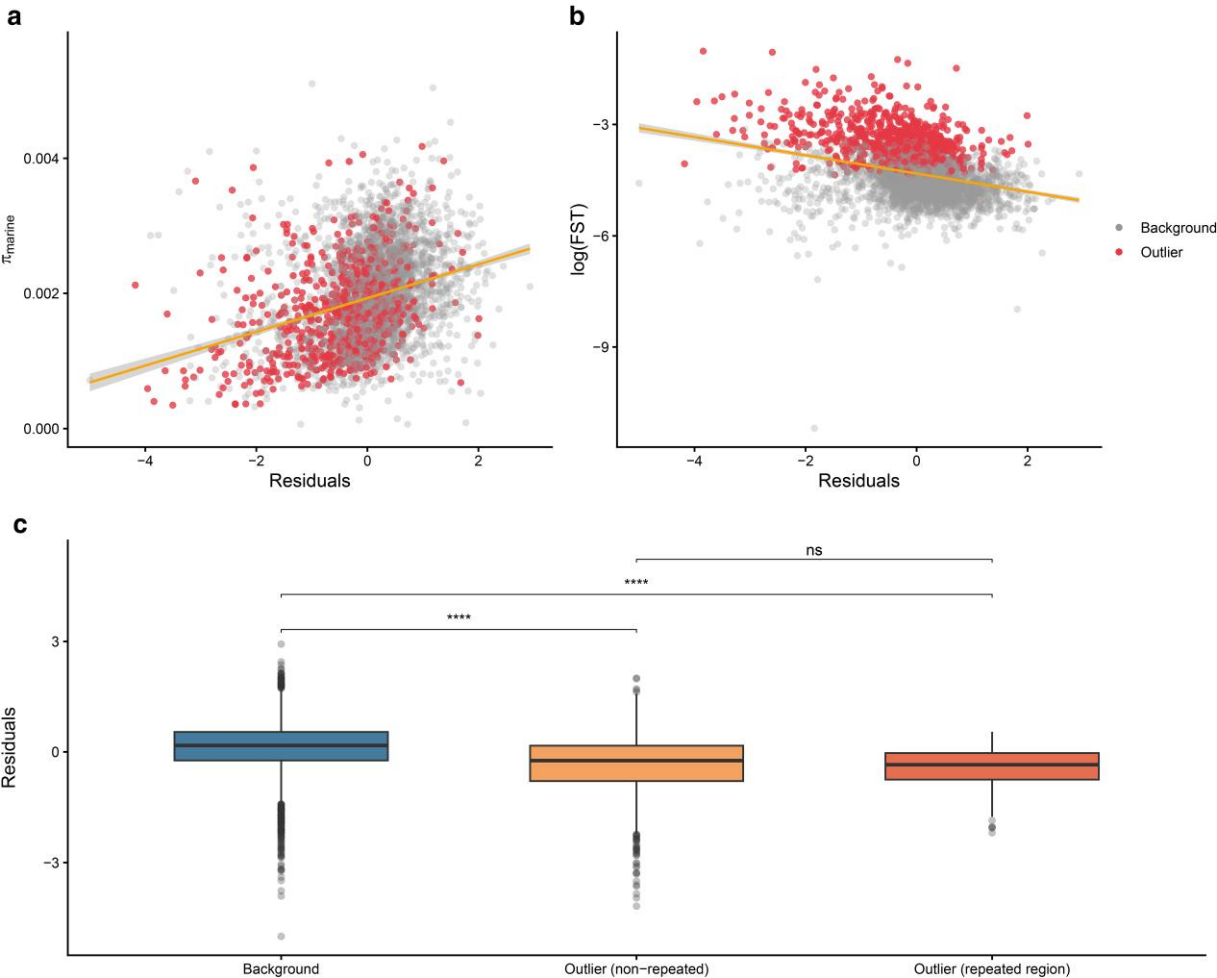
Residuals reveal contrasting correlations with nucleotide diversity (π) and F_ST_. a) Positive correlation between π and residuals in the marine population (Pearson's r = 0.299, *P* < 1 × 10^−78^). Positive residuals indicate regions where observed LD is lower than predicted by the pedigree-based map. b) Negative correlation between marine-freshwater differentiation (log(F∼ST∼)) and residuals (Pearson's r = −0.304, *P* < 1 × 10^−81^). In panels A and B, red points indicate outlier windows and gray points indicate background windows; lines represent the linear regression fitted across all windows. c) Distribution of residuals across genomic categories. Background windows (left) are compared to outlier windows not overlapping repeated regions (center) and outlier windows overlapping repeated regions (right; Roberts Kingman et al. 2021). Both outlier categories exhibit significantly more negative residuals than the background (*P* < 2.80 × 10^−16^), while they do not differ significantly from one another (*P* = 0.329).

Finally, we examined whether residuals from the linear regression between the log-transformed LD-based recombination and the linkage map were associated with π and F_ST_. Residuals were positively correlated with π in both freshwater (Pearson's r = 0.295, *P* < 1 × 10^−76^) and marine populations (Pearson's r = 0.299, *P* < 1 × 10^−78^, Fig. 5a). This indicates that high-diversity regions exhibit lower LD than predicted by the recombination map. In contrast, residuals were negatively correlated with F_ST_ between freshwater and marine populations (Pearson's r = −0.304, *P* < 1 × 10^−81^, Fig. 5b), suggesting that regions of high differentiation exhibit higher LD than expected relative to the recombination map. Comparison of these regions revealed that outlier windows overlapping repeated regions and outlier windows unique to our system both exhibited significantly lower (negative) residuals than background windows. Both outlier windows overlapping repeated regions (mean = −0.462) and outlier windows not overlapping repeated regions (mean = −0.396) showed significantly reduced residuals compared to background windows (mean = 0.118; overlapping: t = 9.47, *P* = 2.80 × 10^−16^; non-overlapping: t = 14.19, *P* < 2.2 × 10^−16^; Fig. 5c), meaning that these regions are enriched for higher LD than expected based on their meiotic rate of recombination. Outlier windows overlapping and not overlapping repeated regions did not differ significantly from one another (t = 0.98, *P* = 0.329).

### Structural variants impact the recombination landscape

We identified a total of 37,935 SVs distributed across the genome, comprising 19,790 deletions, 18,103 insertions, and 42 inversions. Deletions had a mean length of 216 bp (range: 142 to 24,911 bp), while insertions were slightly larger on average (mean = 292 bp, range: 79 to 15,792 bp). Inversions were less frequent but considerably longer, with a mean size of 3,117 bp and lengths ranging from 47 to 28,118 bp.

To identify habitat-associated SVs, we calculated F_ST_ between marine and fluvial populations and defined outliers as those exceeding the 99th percentile of the F_ST_ distribution. This resulted in 386 outlier SVs, comprising both deletions and insertions. No inversions met the outlier threshold, likely due to their limited sample representation.

To assess whether outlier SVs were disproportionately located within highly differentiated genomic regions, we tested their overlap with outlier windows identified with SNPs. A large majority of outlier SVs (78.2%) overlapped with at least one outlier window, compared to 21.8% of background SVs. This enrichment was highly significant (*P* = 2.35 × 10^−121^) with an odds ratio of 12.90, indicating that outlier SVs were nearly 13 times more likely to occur within regions of elevated genetic differentiation based on SNPs. A similar pattern was found for small (odds ratio = 13.32, *P* = 1.57× 10^−115^) and large SVs (odds ratio = 6.93, *P* = 2.42×10^−6^). This enrichment pattern was not driven by variation in SV density across the genome, as SV density within 100 kb windows showed only a very weak association with enrichment significance (Spearman ρ = −0.046, *P* = 0.0028). Thus, the preferential localization of outlier SVs within highly differentiated regions cannot be explained by local SV abundance. Moreover, SVs covered 2.07% of the repeated regions, with 1,681 SVs identified across these loci, including 920 insertions, 759 deletions, and two inversions on chromosome I and VII. Of those overlapping SVs, 56 of them were outlier SVs.

We modeled LD (r^2^) around SVs using linear mixed-effects models that included SV type, distance-weighted SNP separation, and interval between flanking SNPs as fixed effects, with chromosome as a random effect to account for chromosome-level variation. For retained SVs, the median distance from an SV breakend to the nearest flanking SNP was 310 bp, with broadly similar distributions across SV classes (Supplementary Fig. 5). In the freshwater dataset, the interval between flanking SNPs was significantly associated with r^2^ (β = 0.0085, *t* = 12.97), while the effect of frequency-weighted distance was negligible (β = 4.6 × 10^−5^, *t* = 0.07). Inversions were associated with significantly higher LD compared to both deletions (Δr^2^ = 0.0519, *P* = 0.0317) and insertions (Δr^2^ = 0.0532, *P* = 0.0266), as indicated by pairwise comparisons of estimated marginal means. LD around deletions (mean r^2^ = 0.0518) and insertions (mean r^2^ = 0.0505) did not differ significantly (*P* = 0.5847). In the saltwater dataset, the interval between flanking SNPs also had a strong positive effect on r^2^ (β = 0.0094, *t* = 15.42), while distance had no significant effect (β = −0.00020, *t* = −0.33). Although LD around inversions appeared elevated (mean r^2^ = 0.0626) relative to deletions (0.0398) and insertions (0.0380), these differences were not statistically significant (all *P* > 0.30).

We also looked at a potential relationship between the presence of a SV and hotspots and coldspots of recombination. We found that population-specific coldspots contained 232 SVs (enrichment ratio = 2.5, *P* = 0.0009), whereas shared coldspots had 47 overlapping SVs (ratio = 2.34, *P* = 0.0009). Similarly, population-specific hotspots contained 37 SVs (ratio = 0.96, *P* = 0.61), whereas shared hotspots had 16 SVs (ratio = 1.38, *P* = 0.12).

## Discussion

Understanding how recombination landscapes and structural variation interact to shape the genomic architecture of local adaptation under gene flow is central to explaining how adaptive divergence persists in nature. In this study, we compared two geographically close but ecologically distinct populations of three-spined sticklebacks in the St. Lawrence Estuary to test how recombination variation influences the retention of locally adaptive alleles. We found that recombination landscapes varied between populations, revealing population-specific patterns rather than a shared genomic template. Contrary to expectations that local adaptation should be enriched in low-recombination regions, adaptive divergence occurred across regions of varying recombination rates. These regions were disproportionately overlapping known globally repeated adaptive regions identified by Roberts Kingman et al. (2021). Specifically, regions of repeated local adaptation exhibit significantly higher LD than predicted by the pedigree-based recombination map, suggesting that selection—rather than just neutral processes—is actively shaping linkage disequilibrium (which potentially further facilitates multi-locus adaptation, although it is not possible to test this directly here). SVs, particularly insertions, deletions and inversions, emerged as key modifiers of these recombination landscapes, influencing where and how adaptive regions are maintained.

Overall, our findings highlight the complex interplay between recombination, structural variation, and selection in shaping adaptive divergence between connected populations, and underscore the value of integrating recombination mapping with SV analyses to understand the genomic basis of local adaptation in natural populations.

### Recombination landscapes diverge between populations

We found that, despite higher overall recombination rates in the marine compared to the fluvial populations of three-spined stickleback, the overall structure of the recombination landscape remains largely conserved, with genome-wide recombination rates showing strong correlation between populations (r ≈ 0.8). Under LD-based recombination inference, higher recombination rates can be expected in populations with higher genetic diversity, due to reduced background LD (Raynaud et al. 2023). Although genome-wide nucleotide diversity did not differ significantly between our populations, fine-scale variation in diversity may still contribute to these observed differences in recombination rates, as suggested by our model of genetic differentiation. Recombination rate is also expected to scale positively with Ne. However, contemporary Ne values inferred by SMC++ should not be interpreted as literal effective population size since SMC++ assumes panmixia and that violations of this assumption are well known to inflate inferred Ne by increasing the variance in coalescent times (Mazet et al. 2016). The five times higher Ne inferred for the fluvial population likely reflects higher connectivity within the Fluvial Estuary compared to the Marine. Consequently, while demographic differences may influence recombination rates, the subtle but significant divergence detected via the PRDI metric remains robust. Indeed, since migration is known to inflate PRDI values (Talbi et al. 2025), our estimates which assume no migration, likely represent a conservative assessment of the true divergence in recombination landscapes between these partially connected populations.

As demonstrated by Venu et al. (2024), male and female three-spined sticklebacks exhibit substantial differences in their recombination profiles, with sex explaining up to 53% of the observed variation in recombination rate. Importantly, sex and ecotype interact to shape recombination variation: when recombination differs among males, freshwater males exhibit higher recombination rates than marine males, whereas the opposite pattern is observed in females. In the present study, recombination maps were constructed using a balanced number of males and females. Consequently, sex-specific recombination differences that act in opposite directions between ecotypes may partially cancel out when averaged across sexes. This sex-dependent antagonism in recombination differentiation provides a plausible explanation for the absence of strong local Δr outliers between populations, despite evidence for recombination landscape divergence captured by PRDI.

The most dynamic aspect of the recombination landscape appeared at fine scales. Only 19% of recombination hotspots were shared between populations, suggesting rapid hotspot turnover—a pattern previously reported in sticklebacks (Shanfelter et al. 2019) and consistent with recent findings in three salmonid species (Raynaud et al. 2025). This high turnover is thought to stem from the inherently unstable nature of hotspot determinants, such as sequence motifs or chromatin accessibility (Jeffreys and Neumann 2009).

### Interaction between genomic differentiation and recombination

Candidate outlier regions identified through our recombination-informed enrichment framework encompassed ∼0.02% of the genome. These regions were distributed evenly across recombination bins, indicating that elevated differentiation was not strongly biased toward a particular recombination level. While theoretical models suggest that low recombination regions should facilitate the buildup of differentiation by reducing the homogenizing effect of gene flow and preserving locally beneficial haplotypes (Tigano and Friesen 2016; Marques et al. 2019), differentiation can still occur without such facilitation, and these observations show that any such effect may be weak or subtle in natural populations.

The lack of such a pattern in our data likely reflects the polygenic and functionally diverse nature of freshwater adaptation in three-spined sticklebacks. This process involves a broad suite of traits, including morphology, behavior, respiration, and predator defense, highlighting its functional diversity (Schluter 1993; Taugbøl et al. 2020; Aguirre et al. 2022). As a result, selection can act across numerous genomic regions, potentially under different selection intensities and histories. Strongly selected alleles that arose early in the adaptive process may now be found in regions with a high recombination rate, where the benefits of recombination (such as reducing linkage with deleterious mutations) outweigh the advantages of maintaining linkage with co-adapted alleles (Otto and Feldman 1997; Charlesworth 2012). In contrast, more recently selected alleles or those involved in complex epistatic interactions may be preferentially retained in low recombination regions, where linkage preserves beneficial allele combinations (Parée et al. 2025). This duality could explain the lack of a simple relationship between recombination and outlier enrichment. Alternatively, the influence of recombination on outlier enrichment may be relatively weak compared to the strength of selection. If adaptation requires changes at specific loci, these targets can still accumulate adaptive mutations even when they fall in regions of higher recombination, leading to detectable signals of differentiation irrespective of local recombination context.

These findings also align with the concerns raised by Booker et al. (2020), who warned that ignoring recombination rate heterogeneity in genome scans could lead to biased inference of selection. Specifically, F_ST_ distributions tend to have long tails in low recombination regions, increasing the propensity to fall outside of any threshold based on genome-wide values, with the opposite pattern in high recombination regions due to their tighter distributions. Our results confirm this pattern, underscoring the importance of accounting for recombination when interpreting genome-wide scans of differentiation.

The observed interaction between nucleotide diversity (π) and LD-based recombination rate (ρ) in our F_ST_-based models highlights another fundamental challenge in interpreting genomic regions of differentiation. In our dataset, the correlation between π and F_ST_ was significantly higher in regions of low recombination. This likely reflects the impact of linked selection, where both background selection and selective sweep reduce local diversity. Consequently, regions of low diversity often exhibit elevated LD, leading to downward-biased recombination estimates, whereas regions of high diversity produce less biased but noisier estimates. This suggests that F_ST_ could partly reflect underlying differences in within-population genetic diversity, especially in low recombination regions where both linked selection and ancient coalescent events can exacerbate differentiation (Noor and Bennett 2009; Cruickshank and Hahn 2014). Comparable links between recombination, diversity, and differentiation have been observed in Eurasian blackcap where population-specific reduction of recombination biased their detection of potentially adaptive variants (Ishigohoka et al. 2024).

However, our residual analysis highlights how different selection regimes can shape the local genomic landscape. In this analysis, negative residuals represent regions that have a lower recombination rate ρ than expected by the pedigree-based recombination map (Rastas et al. 2016) while positive residuals represent regions that have a higher ρ than expected. We observed a positive correlation between residuals and π, and a negative correlation with F_ST_. These contrasting relationships are expected under both directional and background selection, as both processes result in low diversity and high LD by purging genetic variation and extend the reach of linked selection (Charlesworth et al. 1997; Kaushik 2023). While these processes produce overlapping genomic signatures that are difficult to disentangle entirely, our results suggest they reduce the effective recombination rate in regions of high differentiation and low diversity. This localized suppression of effective recombination, evidenced by our negative residuals, likely plays a critical role in shaping the architecture of divergence by maintaining linkage across multiple sites. Such a landscape could further facilitate multi-locus adaptation by preserving co-adapted allelic combinations, especially in the presence of gene flow (Yeaman and Whitlock 2011).

Altogether, these observations support a more nuanced view of how recombination influences the genomic landscape of divergence. Rather than a uniform bias toward differentiation in low recombination regions, our results suggest that the strength and timing of selection, the haplotype structure of a genomic region, the polygenic nature of adaptation, and variation in genetic diversity all contribute to determine where outliers emerge in the genome.

### Structural variants modulate the recombination landscape

The patterns observed in our study support a role for SVs in shaping the recombination landscape. We found that SNP pairs for which a larger proportion of their distance were spanned by an SV exhibited elevated LD, consistent with recombination suppression across multiple SV categories. In addition, recombination coldspots were enriched in SVs, further reinforcing their potential role in modifying the recombination landscape. Similar patterns have been reported elsewhere: Morgan et al. (2017) found that recombination coldspots in mice often coincided with CNVs, while Huang et al. (2025) showed that transposable elements generating structural variation also reduced recombination rates in their vicinity in *Drosophila melanogaster*. Together, these findings reinforce the idea that SVs may interfere with chromosome pairing or recombination initiation, which may be driven by both structural constraints and epigenetic mechanisms (Casale et al. 2024; Johnston 2024).

The strongest effect of SVs on recombination was detected for inversions, which were associated with substantially elevated LD and thus likely reduced effective recombination rates. This relationship was statistically significant in the fluvial population, while a similar but weaker trend was observed in the marine population. On the technical side, types of SV differ in how they affect SNP calling and LD-based inference (Stuart et al. 2026). Inversions are comparatively robust because the inverted segment remains fully present in both haplotypes, preserving read depth and mappability, whereas insertions and deletions alter local copy number and sequence content. As a result, SNPs overlapping heterozygous deletions may be miscalled as homozygous, and SNPs tagging insertions may be preferentially removed by standard depth-based quality filters. These effects can reduce marker density and add noise to LD estimates around CNVs, potentially dampening their apparent association with recombination suppression relative to inversions.

Differences between populations may also reflect interactions between technical and demographic factors. The larger sample size of the marine population likely increased the precision of LD estimates but may also have amplified the effects of subtle population substructure, which can inflate LD through allele frequency correlations even in the absence of reduced recombination (Li and Nei 1974; Ohta 1982). Such background LD can obscure or dilute the detectable impact of SVs on recombination rate inference. Biological factors, including inversion age, size, and associated haplotype composition, are also known to influence the strength of recombination suppression (Stevison et al. 2011; Charlesworth 2023). However, because inversion heterozygosity was similar across populations, these factors alone are unlikely to fully explain the population-specific differences observed here.

Taken together, our results indicate that SVs are important contributors to recombination rate variation and can shape patterns of genetic variation in both connected and partially isolated populations. Inversions showed the strongest and most consistent recombination-suppressing effects, likely reflecting both their biological impact and their already known relative robustness to SNP-calling artefacts. Nevertheless, other SV types should not be overlooked. Experimental work has demonstrated that insertions and deletions can directly reduce recombination efficiency at the molecular level by disrupting sequence homology (Brenner et al. 1985), and comparative studies have linked CNVs and intrachromosomal rearrangements to recombination rate variation in natural populations (Völker et al. 2010). Although the effects of insertions and deletions were more modest in our dataset, these findings underscore the importance of considering the full spectrum of SVs and the technical limitations of SNP-based inference when interpreting the genomic architecture of recombination landscapes.

### Concentrated genomic architecture of local adaptation in the presence of gene flow

The detection of repeat regions shared between our dataset and the globally reused targets of adaptation reported by Roberts Kingman et al. (2021), together with previous work in the St. Lawrence Estuary by McCairns and Bernatchez (2008), supports the presence of local adaptation despite ongoing gene flow. McCairns and Bernatchez (2008) showed that fluvial and marine sticklebacks in this system form distinct genetic groups and that environmental rather than geographic factors explain a large proportion of their differentiation, highlighting the role of ecological selection in maintaining divergence under migration. Our results extend this view by demonstrating that several of the same genomic regions involved in global freshwater adaptation also contribute to divergence in the St. Lawrence Estuary. This reuse of previously identified globally repeated targets of local adaptation is consistent with the species’ well-documented capacity for rapid adaptation to freshwater (Barrett et al. 2011; Terekhanova et al. 2014). At the same time, our results highlight a suite of genomic regions unique to our system, which may reflect ecological differences in the St. Lawrence Estuary compared to more typical freshwater–marine contrasts. For instance, unlike many freshwater stickleback populations, our fluvial fish retain extensive armor, likely due to the persistence of predators in the estuary (García-Machado et al. 2022) as it is the case for other estuarine stickleback populations from western Canada (Reimchen 2000). Nonetheless, other traits such as feeding ecology, reproductive behavior, and aspects of morphology do differ between environments (Mccairns and Bernatchez 2012), as do abiotic variables such as pH, salinity, and temperature. Genes associated with these traits are possibly those contributing to the repeated signals we observed.

Repeated regions were not randomly distributed across the genome. Instead, more than half (54%) of them were concentrated on three chromosomes (IV, VII, and XXI), which we designated as major hotspots of local adaptation in this system. This pattern is consistent with previous reports of concentrated architectures in stickleback (Hohenlohe et al. 2010; Jones et al. 2012; Roberts Kingman et al. 2021). These regions may represent clusters of tightly linked adaptive loci, as suggested by their reduce recombination rates compared to non-repeated outlier windows, or they may house highly pleiotropic genes under strong selection. Such a combination of clustered and distributed patterns is in line with theoretical expectations under migration-selection balance, where selection maintains large-effect loci in low recombination regions while smaller-effect loci are distributed more broadly across the genome (Yeaman and Whitlock 2011; Yeaman 2022). Indeed, a more concentrated architecture of local adaptation was found in *Populus trichocapra* populations that were subject to higher gene flow compared to more distant populations (Holliday et al. 2016).

Interestingly, none of the 42 inversions we genotyped showed differentiation high enough to qualify as outliers, an unusual result given the prominence of inversions in many recent studies of adaptation with gene flow (Shi et al. 2023; Fuentes-Pardo et al. 2024; Sabatino et al. 2025). This may indicate that inversions in our system are relatively young, as suggested by their lower F_ST_ and minimal variation in nucleotide diversity (Akopyan et al. 2025 ). Still, their potential role cannot be ruled out as two inversions, on chromosomes I and VII, overlapped repeated adaptive regions. The former has been previously linked to freshwater adaptation in sticklebacks (Rodríguez-Fuentes et al. 2025), while the latter, associated with defense trait QTL (Peichel and Marques 2017), is a novel candidate in our system. Notably, we did not detect the known inversions detected in the other three-spined stickleback systems on chromosomes XI and XXI (Jones et al. 2012; Roesti et al. 2015).

Overall, recombination landscapes and SVs, not only inversions but also insertions and deletions, influence local patterns of recombination and differentiation. Clusters of repeated adaptive regions coincide with some SVs and low-recombination areas, highlighting how these genomic features contribute to the organization of adaptive variation in connected populations.

## Supplementary Material

jkag086_Supplementary_Data

## Data Availability

Raw sequence reads come from Delaive et al. (2025) study and are deposited in the SRA (BioProject PRJNA1327819). SVs and SNPs vcf file can be found at Dryad (https://doi.org/10.5061/dryad.cc2fqz6md). Source code for the creation of recombination maps can be found at Github (https://github.com/Sannouche/Recombination), and the code for outlier detection can be found at Github (https://github.com/Sannouche/Outlier_Detection). Supplemental material available at G3 online.
