# Supplementary material for: The genomic architecture of local adaptation in two connected populations of three-spined stickleback
Source: G3 (Bethesda). 2026 Apr 1;16(6):jkag086. doi: 10.1093/g3journal/jkag086 (PMC13232512; doi:10.1093/g3journal/jkag086)
Supplement: jkag086_Supplementary_Data [file jkag086_supplementary_data.docx]

**F**
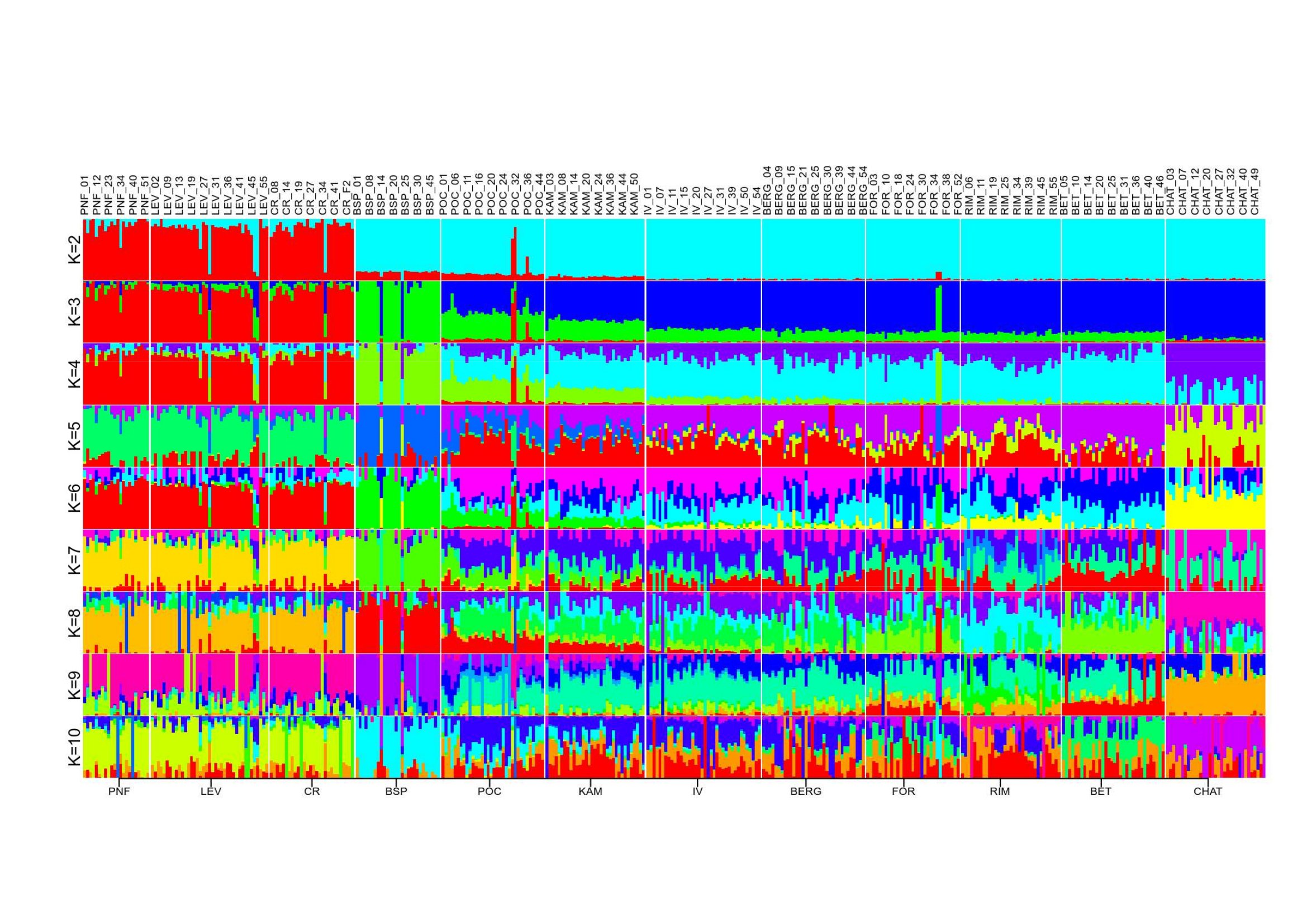
**igure S.1:** Assessment of population genetic structure and ancestral proportions. Ancestry proportions inferred by ADMIXTURE for K = 2 to K = 10. Individuals (columns) are ordered geographically from the Fluvial Estuary (right) to the outer Marine Estuary (left). At the optimal cross-validation (CV) error (K = 2), a clear separation is observed between the primary fluvial and marine clusters. Increasing K values reveal finer-scale substructure, notably the genetic distinctiveness of the Baie-Saint-Paul population (K = 3). Based on these results and prior evidence of distinct evolutionary history (Delaive et al. 2025), Baie-Saint-Paul individuals were excluded from downstream recombination comparisons.


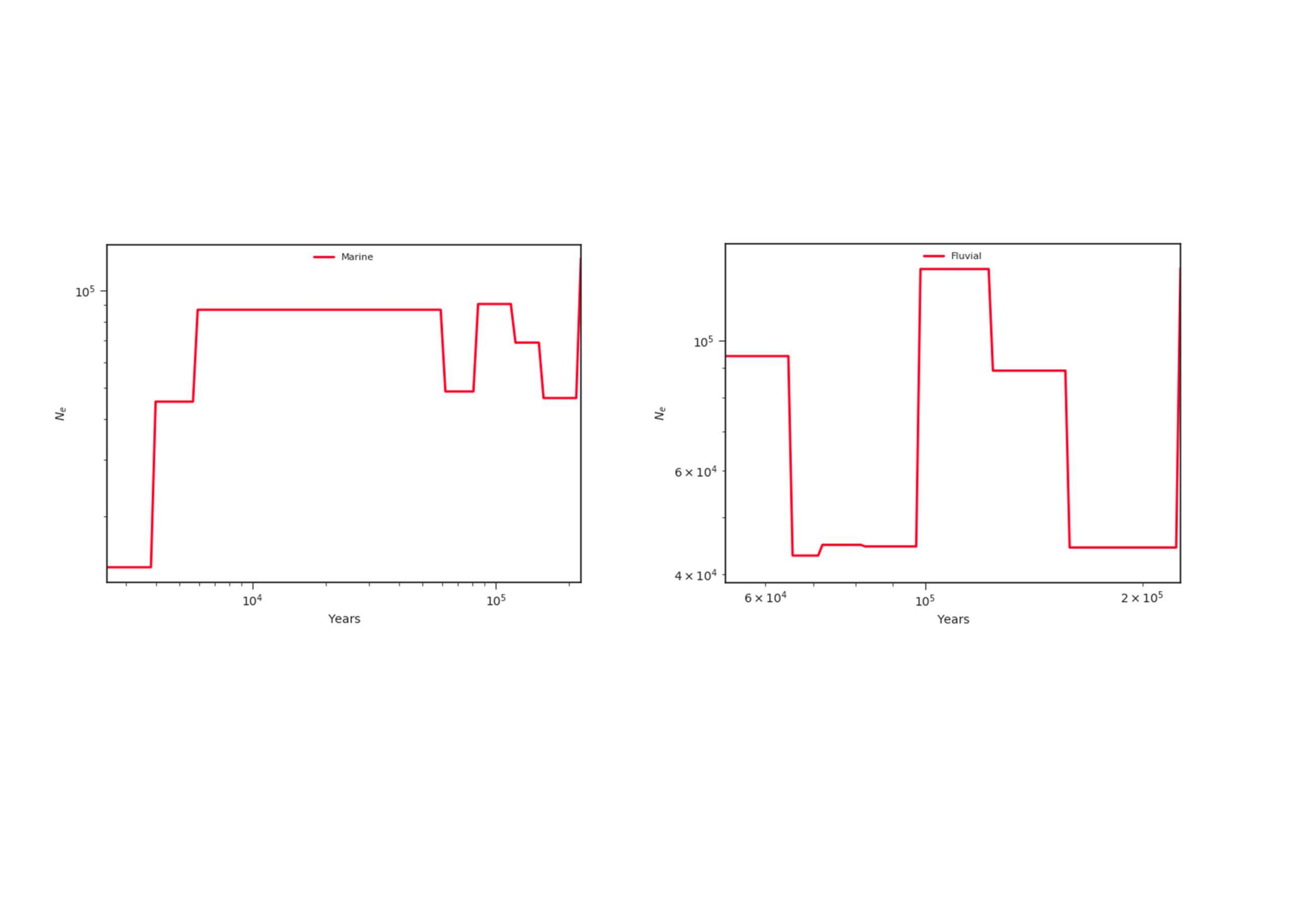
**Figure S.2:** Historical demographic trajectories of marine and fluvial stickleback populations. Effective population size (Ne) through time inferred by SMC++ across the chromosome I. The x-axis represents the time in generations before present, and the y-axis represents the inferred Ne. Results are shown separately for the fluvial population (left panels) and the marine population (right panels).


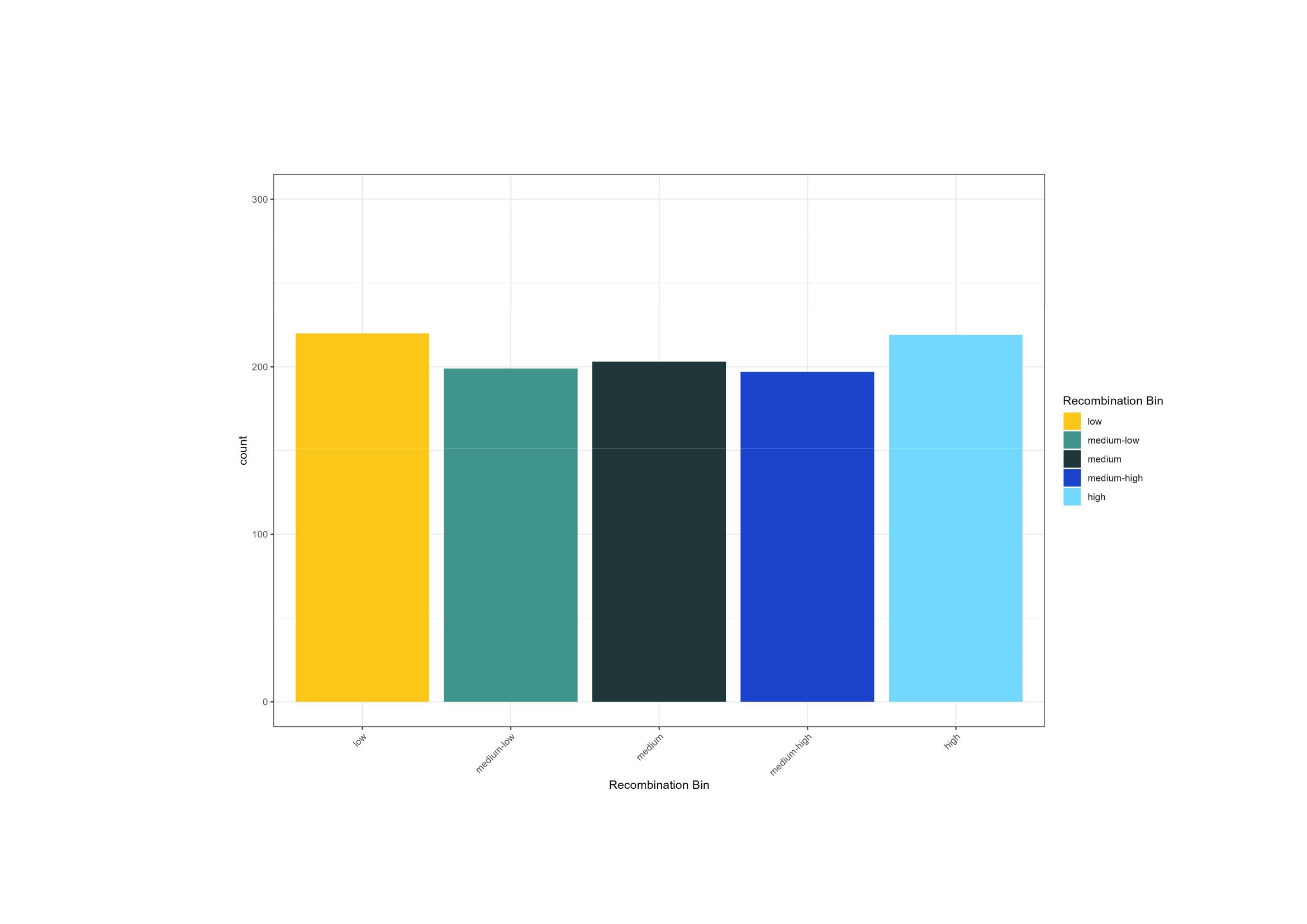


Figure S.3: **Distribution of outlier windows across recombination bins.** Bar plot showing the frequency of outlier windows (n = 553) identified through the recombination-informed enrichment framework across five recombination rate categories (ranging from low to high). A chi-squared test confirms that outliers are distributed evenly across the recombination landscape (p-value = 0.66), representing approximately 0.02% of the genome.


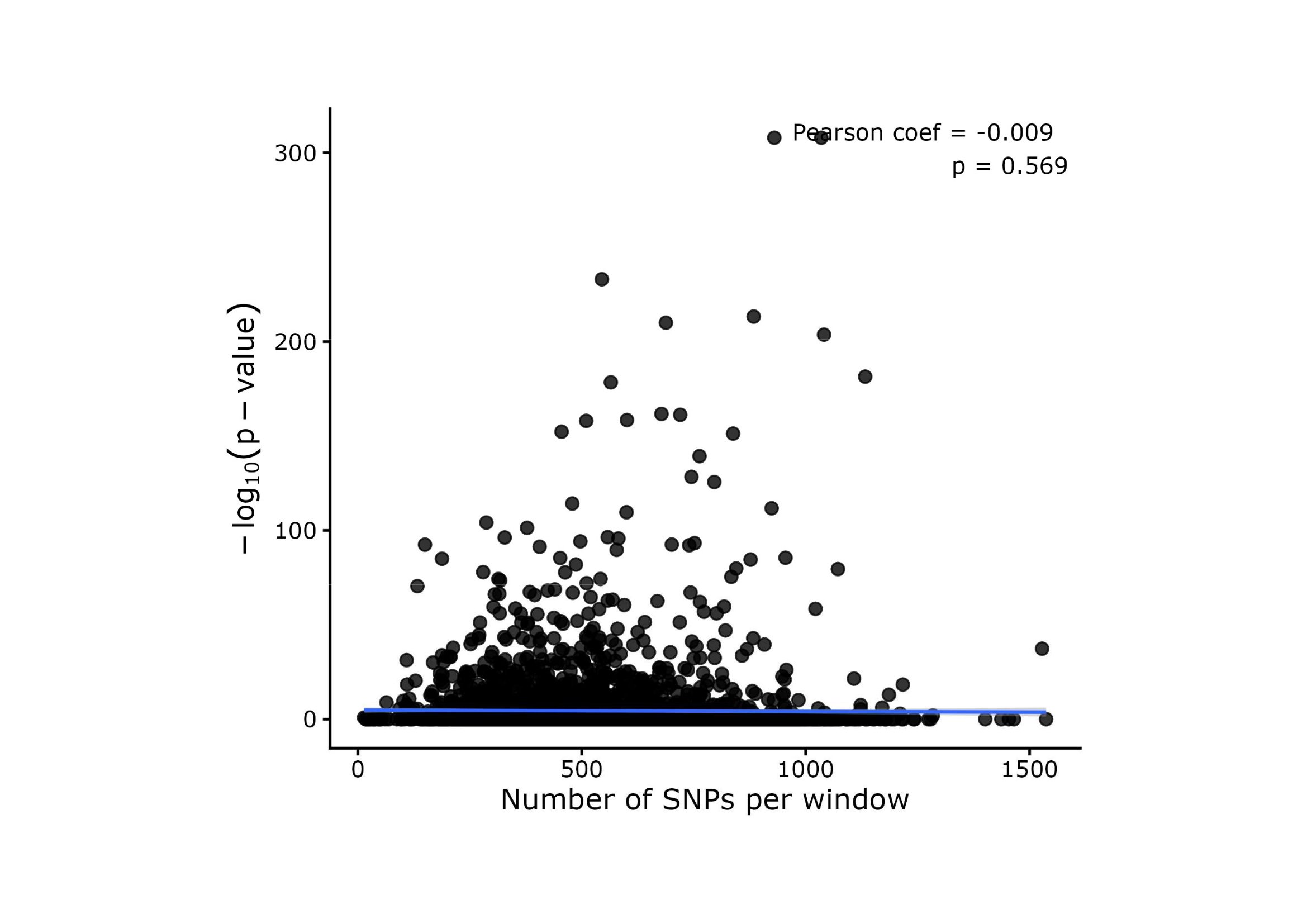
**Figure S.4:** **Independence of outlier enrichment from local SNP density.** Scatter plot illustrating the relationship between the number of SNPs per 100-kb window and the –log_10_-transformed non-exact p-value from the recombination-informed enrichment test. The blue line represents a linear regression fit (*geom_smooth*), indicating a non-significant correlation (Pearson’s coefficient = -0.009, p-value = 0.569).


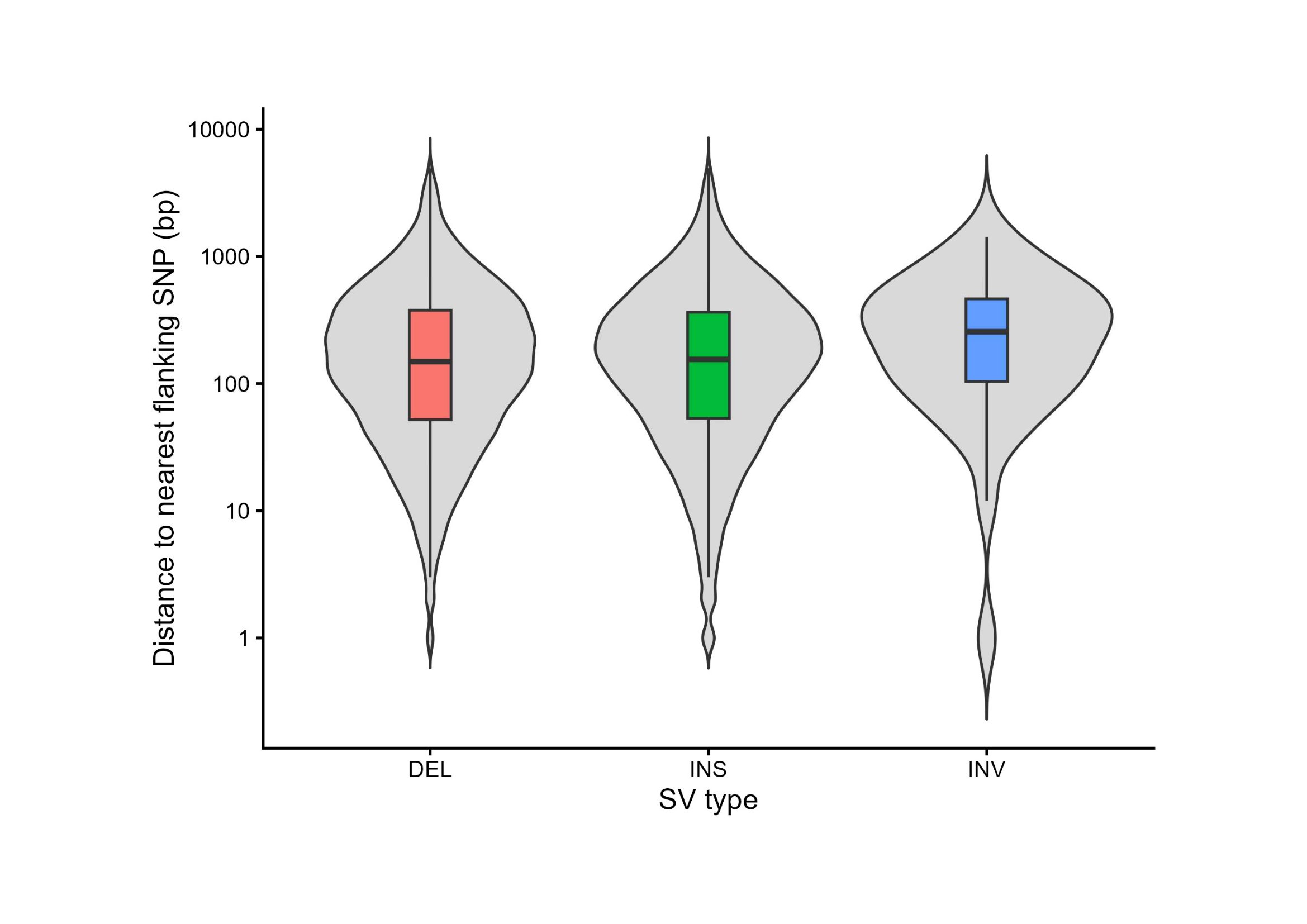
**Figure S.5:** Genomic proximity of flanking SNPs to structural variant (SV) breakends. Violin plots illustrating the distribution of physical distances (bp) between SV breakends and the nearest flanking SNPs utilized for linkage disequilibrium modeling. Data are categorized by SV type: deletions (DEL; red), insertions (INS; green), and inversions (INV; blue). The median distance to the nearest flanking SNP across all retained SVs was 310 bp, with consistent distributions observed across all structural classes.
